# The Role of Viral Pathogens in Horse Respiratory Diseases: A Cytological and Molecular Approach Using Next-Generation Sequencing

**DOI:** 10.3390/ani14233347

**Published:** 2024-11-21

**Authors:** Maurizio Mazzei, Benedetta Sorvillo, Micaela Sgorbini, Francesca Bindi, Alice Perelli, Fulvio Laus

**Affiliations:** 1 Department of Veterinary Sciences, University of Pisa, Viale delle Piagge 2, 56124 Pisa, Italy; maurizio.mazzei@unipi.it (M.M.); benedetta.sorvillo@vet.unipi.it (B.S.); micaela.sgorbini@unipi.it (M.S.); perelli.alice95@gmail.com (A.P.); 2 School of Biosciences and Veterinary Medicine, University of Camerino, Via Pontoni 5, 62032 Camerino, Italy; fulvio.laus@unicam.it

**Keywords:** horse, bronchoalveolar lavage fluid (BALF), metagenomic next-generation sequencing (NGS)

## Abstract

(1) Background: Respiratory problems may be associated with pathogens among which viruses may play an important role in causing or promoting clinical signs. However, traditional diagnostic methods for equine infectious diseases, such as PCR, are limited to known pathogens and often miss rare or novel viruses. Metagenomic next-generation sequencing is a transformative method that allows the sequencing of all genomes present in a sample. Recent studies have used next-generation sequences to identify viral sequences in horses with unexplained respiratory symptoms, revealing potential links between viruses and respiratory problems. The aim of the study was to verify a relationship between respiratory diseases and the presence of viral agents through molecular analyses, applied to bronchoalveolar lavage fluid obtained from horses with history or presence of clinical signs of respiratory diseases. (2) Methods: A cohort of 14 horses was enrolled. All the subjects were submitted to a clinical assessment of the respiratory tract, thoracic ultrasound evaluation, respiratory tract endoscopy, and bronchoalveolar lavage. The latter one was used for cytological analysis, DNA and RNA extraction, and molecular biology analysis. (3) Results: No positive results were obtained in the molecular studies except for a sequence of 753 bp obtained by next-generation sequences, with complete homology to Equid gammaherpesvirus 2 strains. The samples were taken from a thoroughbred female horse aged 2 years old and referred for poor performance and sporadic cough at the beginning of the training session. (4) Conclusions: viral involvement may be not common in horses with respiratory diseases. We cannot exclude that the lack of virus detection may be due to the small sample size of the population included.

## Simple Summary

The aim of this study was to verify a relation between respiratory diseases and the presence of viral agents through molecular analyses. A cohort of 14 horses was enrolled over a 6-month period. All the subjects underwent clinical evaluation of the respiratory tract and to a bronchoalveolar lavage used for cytological analysis, and molecular biology analysis. No positive results were obtained in the molecular studies carried out by both PCR and NGS methods, except for a sequence of 753 bp obtained from a thoroughbred female horse aged 2 years old and referred for poor performance and sporadic cough at the beginning of the training session. Sequence analysis resulted in complete homology to Equid gammaherpesvirus 2 strains. Viral involvement did not seem to be common in horses with asthma. The lack of virus detection may be due to the small sample size of the population included.

## 1. Introduction

Poor performance refers to a condition in which a horse, despite adequately training, is no longer able to achieve past and expected results. This syndrome can present with a range of clinical symptoms, such as an unwillingness to work, exercise intolerance, and a decreased performance in specific athletic tasks [1].

This condition is frequently not associated with clinical symptoms caused by specific pathologies, making the diagnosis challenging. In this regard, it fundamental to collect the subjects’ medical history as comprehensively as possible, and a thorough evaluation of multiple body systems is often needed. This requires carefully directing any specialist collateral investigations. The symptoms depend on the horse’s sporting discipline; for example, racing horses may exhibit sudden slowdowns, and show jumping horses can make mistakes during competitions that they performed correctly in the past [2]. The causes of poor performance are classified based on the system involved, and the respiratory system is secondary only to orthopedic pathologies [3]. Concerning the lower respiratory tract, mild equine asthma (MEA) and exercise induced pulmonary hemorrhage (EIPH) are frequently diagnosed in horses with poor performance, cough, and normal breathing at rest [4,5]. On the other hand, severe equine asthma (SEA) usually causes exercise intolerance, regular to frequent coughing, and increased respiratory efforts at rest. The clinical signs improve with strict environmental control and treatment (i.e., corticosteroids and bronchodilators) [4].

The study of infectious diseases in horses has been essential to veterinary science. Over the past century, many equine viral diseases and their causes have been identified using traditional virology, but the origins of other equine diseases remain unknown [6].

Modern virus diagnostics primarily rely on polymerase chain reactions (PCR), yet they are limited to detecting only the pathogens specified in the assay. This means that rare or mutated pathogens with atypical clinical presentations, as well as new and unknown ones, may go undetected [7].

Next-generation sequencing (NGS) is a technique for detecting all genomes in a sample. Since the early 2000s, new sequencing technologies characterized by high speed and throughput have been introduced, allowing the concurrent sequencing of large numbers of samples and the identification and classification of several agents in a single sample [7]. The use of these new technologies eliminates the need for initial knowledge of the genomic sequences present in the sample, and offers multiple advantages compared to traditional methods such as PCR which, on the contrary, depends on specific primers [8].

To date, NGS technologies have been decisive for the discovery of new viruses and the characterization of viral communities. They are used for the sequencing of entire genomes and the investigation of variability within the genome, epigenetics, metagenomics, and gene-expression profiling. In microbiology and virology, NGS is used to analyze the variability of the viral genome within a host and to identify drug-resistance mutations [9].

In a recent study [10], metagenomics was applied for identifying viral sequences in the plasma and respiratory swabs of equine with respiratory symptoms that could not be explained. Two copiparvoviruses and equine pegivirus D were detected in the plasma of horses displaying respiratory symptoms, while equine herpesviruses 2 and 5 were detected in respiratory swabs. These findings were substantially linked to respiratory symptoms in this sample. The present study aims to verify a relation between respiratory diseases and the presence of viral agents through molecular analyses, applied to bronchoalveolar lavage fluid (BALF) obtained from horses with history or presence of clinical signs of respiratory diseases.

## 2. Materials and Methods

### 2.1. Animals

This observational study was performed over a 6-month period after receiving approval by the Research Ethical Committee of the University of Pisa (DL 26/2014, article 2, paragraph 1, letter b. Research Ethical Committee of the University of Pisa; approval number n. 3, date 22 January 2021) and an owner oral consent for clinical procedures.

A cohort of 14 horses with history of respiratory signs (cough, nasal discharge) in the last 1 month or presence of respiratory signs, poor performance, or exercise intolerance, underwent a respiratory tract assessment on the owner’s request. All horses were vaccinated against tetanus and influenza and 10 out of 14 against herpesvirus 1 and 4 following the AAEP guidelines [11]. All horses underwent a complete clinical exam, thoracic ultrasound assessment, endoscopic examination at rest of the respiratory tract, and bronchoalveolar lavage (BAL).

### 2.2. Thoracic Ultrasound, Respiratory Endoscopy, BAL, and BALF Cytological Analysis

The thoracic ultrasound was performed as previously reported [12] on all the subjects not sedated, but just manually restrained using a portable machine (MyLab SigmaVET, Esaote, Firenze, Italia) and multifrequency probe.

The respiratory endoscopy and the BAL were performed after at least 8 h of fasting and under sedation with alfa_2_-agonist (detomidine, 0.01 mg/kg ev), as previously reported [12,13]. Respiratory endoscopy was always performed before BAL execution using a 120 cm video endoscope and a portable processor (Storz, Tuttlingen, Germany). The endoscope was introduced blindly through the nasal passage and then into the trachea. The respiratory tract was visualized, and mucus found in the trachea was scored as previously proposed [14].

After endoscopy, the BAL was performed using a commercially available equine BAL catheter (Bivona Inc., Gary, IN, USA), introduced as previously described for endoscopy. To desensitize the tracheal lumen while advancing the tube for antitussive purposes, a solution of lidocaine diluted at a concentration of 0.66% with sterile 0.9% saline was used. The BAL catheter was advanced to the third and fourth generation of bronchi to sample a wide portion of the lung and prevent discrepancies in the evaluation of the cells differential count. The cuff was then inflated with air, and 300 mL of warm, sterile, isotonic, and crystalloid solution was infused. Samples were collected with 60 mL syringes by manual aspiration of at least 40% of the infused volume. Finally, the cuff was deflated, and the tube was removed. The BALF was divided in two aliquots: one aliquot was collected in ethylenediaminetetraacetic acid (EDTA) tubes to reduce cell clumping and processed within 1 h from the collection for cytological analysis, and the second aliquot was stored in a sterile PVC tube and preserved at −80 °C for DNA and RNA extractions.

An automated counter equipment (Hecovet, SEAC-RADIM Co, Florence, Italy) was used to assess the total nucleated cell count (TNCC) of the BALF on EDTA samples. Slide preparation was conducted by cytocentrifugation (Cytofuge 2, Statspin, Fairview, PA, USA) with 400 uL per slide and 300 rpm for 10 min. Slides, after air-fixing, were stained with May–Grundwald Giemsa using an automatic colorimeter machine (Aerospray Hematology Slide Stainer mod. 7150, Delcon, Grassobbio, Italy) for a duration of 12 min. For the optimal area coverage, each stained smear was scanned at low magnification (×200), then at higher magnification (×400). Cells were recognized and counted using oil immersion (×1.000). The count process started from the upper edges, progressing towards lower edges to prevent revisiting. A total of 400 cells were/smear were counted for each differential count, using a bright-field optical microscope (Leica DM 2000 LED, Leica Microsytems CSM GmbH, Wetzlar, Germany).

### 2.3. DNA and RNA Extractions

The BALF samples were thawed at 4 °C. A starting volume of 40 mL was then subjected to centrifugation at 2500 rpm at 4 °C for 10 min in a 50 mL Falcon conical tube. The pellet was resuspended in 1 mL of supernatant and stored at −80 °C until used for nucleic acid extraction using “AllPrep DNA/RNA Mini kit” following the manufacturer’s instructions.

DNA was eluted in 50 μL, while 30 μL of elution buffer was used for RNA, and both DNA and RNA were subjected to quantitative and qualitative evaluation using a Qubit (Thermofisher, Waltham, MA, USA) fluorometer. Viral targets for investigation through PCR and RT-PCR analysis were selected based on a literature review that considered viral agents (Equine Rhinitis B, Equine Herpes 1, 4, 5, Bosavirus, Horse Parvovirus CSF, Equicopivirus) identified or researched in similar studies [10,15,16,17,18] (Table 1).

As a control for the proper extraction of nucleic acids, a verification PCR was conducted on the equine GAPDH control gene, for both DNA and RNA assays. From the RNA samples, the ‘M-MLV Reverse Transcriptase’ kit (Invitrogen, Waltham, MA, USA) was used to synthesize complementary DNA. Molecular analyses were performed using the “WONDER Taq Hot Start” kit from the company “EuroClone” (EuroClone, Lombardy, Italy). The final PCR products were visualized through agarose gel electrophoresis.

### 2.4. NGS

To acquire additional information through molecular techniques characterized by a greater depth of analysis, samples were submitted for NGS sequencing. Each nucleic acid sample (DNA and cDNA) underwent quantitative (ng/μL) and qualitative (260/280 absorbance ratio) evaluation using the NanoDrop instrument (Thermofisher Scientific, Waltham, MA, USA). For each sample, 100 ng of nucleic acid with a 260/280 absorbance ratio value < 1.8 was prepared for submission.

Analysis was conducted using next-generation sequencing (NGS) technology employing a SHOTGUN protocol with a depth of analysis set at 30 million reads on the Illumina platform (Illumina, San Diego, CA, USA). The analyses were performed by private laboratory (Genomix4Life Srl, Salerno, Italy). The obtained results were then bioinformatically analyzed by Geneious 2023.1.2 software (https://www.geneious.com accessed on 10 April 2023) and galaxy online platform (https://usegalaxy.org/ accessed on 13 October 2022).

An operational workflow was subsequently established to identify the presence of equine viral genomic sequences. An equine virome database set was generated by the NCBI platform (https://www.ncbi.nlm.nih.gov/labs/virus/vssi/#/ accessed on 5 October 2022). Upon obtaining the sequences of interest aligned with the equine virome, the associated viral targets were identified, and a consensus sequence was generated.

## 3. Results

Based on clinical exam, thoracic ultrasound, respiratory endoscopy, mucus score assessment, and BALF cytology, horses were divided in two groups:

- Group A, healthy horses: 5/14 (35.7%) trotter female horses were included, median age 7 years (6–9 years), median body weight 455 kg (440–585 kg). In those animals, no clinical signs were present at the time of assessment, few B lines on both lungs on thoracic ultrasound, normal respiratory endoscopy, mucus scored 1/5 in all the subjects, BALF cytology within reference ranges [4].
- Group B: 9/14 (64.3%) horses were included, of which 2/9 were gelding and 7/9 female; 5/14 thoroughbred, 1/14 saddlehorse, 3/14 trotters, with a median age of 9 years (2–19 years) and a median body weight of 500 kg (450–540 kg). Clinical signs were present at the time of assessment in 6/9 horses, while no clinical signs were present at the time of assessment in 3/9, few B lines on both lungs on thoracic ultrasound in all the subjects included, respiratory endoscopy was normal in 8/9 horses, while 1/9 was affected by laryngeal lymphoid hyperplasia grade 3/4, mucus was scored from 2/5 to 3/5, BALF cytology was not within reference intervals [4]. The results for group B are summarized in Table 2.

No positive results were obtained in the molecular studies carried out by both PCR and RT-PCR methods, using single and nested protocols against six DNA viral targets (equine herpes 1, 4, 5, bosavirus, equine parvovirus CSF, equicopivirus) and one RNA viral target (equine rhinitis B). However, it is noteworthy that PCRs performed on the GAPDH control gene yielded positive results for all samples used, demonstrating the correct extraction efficiency for both DNA and RNA.

The only sample that showed a sequence of 753 bp, longer than 300 bp, belonged to a thoroughbred female horse aged 2 years old included in group B and referred for poor performance and sporadic cough at the beginning of the training session. This sequence was obtained through NGS analysis, and after sequence assembly resulted in a sequence of 753 bp length with identical sites (100.0%)] and pairwise identity (100.0%), referable to Equid gammaherpesvirus 2 strains (MW322569). The horse showed a low number of B lines revealed with thoracic ultrasound, lymphoid hyperplasia grade ¾, and mucus score 3/5 assessed by respiratory endoscopy, and MEA was diagnosed on BALF cytology assessment (Table 2). The BALF was mildly cloudy, but no fibrin flocs were present.

The results obtained using NGS technology allowed the presence of 19 sequences to be identified in the subject belonging to both group A and B, with 100% homology to two specific sequences: LC193725.1 (equine alphaherpesvirus 1), KX905134.1 (Alcelaphine herpesvirus 1), but all presenting short consensus sequences no longer than 300 bp. It is to be emphasized that only one sample belonging to group B had a score sequence of 753 bp with complete homology to Equid gammaherpesvirus 2 strains (accession number MW322569), which was subsequently shown to be homologous to the sequence associated with the ORF 10 gene, which is involved in the activation of viral transcripts through “immediate early gene transactivation”.

## 4. Discussion

The aim of this study was to verify a relationship between respiratory diseases and the presence of viral agents through molecular analyses, applied to bronchoalveolar lavage fluid obtained from horses with history or presence of clinical signs of respiratory diseases. Overall, the molecular investigations conducted using both conventional PCR and NGS did not reveal the presence of significant viral agents. Using conventional PCR, despite testing all subjects for the main targets of pulmonary pathogens suggested by even the most recent studies [10,16,17,18], no viral infections were detected in either of the examined groups, suggesting that pulmonary viral pathogens are not frequently present. To avoid the potential failure in identifying viral targets due to the specificity of the PCR protocols used, the metagenomic approach was also employed. Viral metagenomics has played a significant role in the recent discovery of various animal viruses [19,20,21]; the shotgun method applied in this study is potentially capable of identifying, in conjunction with an in-depth bioinformatic analysis, any viral genome presents in the sample. Using this approach, the identification in both groups of a total of 19 sequences, showing 100% homology with Equid Alphaherpesvirus 1 (LC193725.1), Alcelaphine Herpesvirus (KX905134.1), initially suggested a viral involvement, but the length of the consensus sequence generated by the de novo assembly resulted in only short sequences (from 170 to 254 bp), which are unlikely to be associated with the presence of an active viral infection. It is also important to know that the detection of short genomic sequences through NGS, associated with viral sequences via BLAST analysis, should be interpreted cautiously, especially when dealing with assembled sequences of extremely short length (<300 bp). However, these short sequences can still suggest the presence of broader viral groups or families. Supporting this hypothesis, we must consider the negative PCR results for specific targets of EHV-1, EHV-4, and EHV-5; in fact, conventional PCR is able to identify sequences belonging only to a limited portion of the viral genome. Next-generation sequencing (NGS) might detect traces of viral genomes independently by primer design that could represent infections that occurred months, or even years, earlier. These past infections may contribute to the development or exacerbation of chronic respiratory conditions, including asthma.

Moreover, the homology observed with Alcelaphine Herpesvirus (KX905134.1) can be considered a nonspecific result, indicating only a presence of a part of the herpes viruses genome. Further, BLAST analysis showed similar and slightly lower E-value scores on other herpesvirus sequences, specifically EHV-1 (accession number LC193725.1), highlighting the lack of specificity when analyzing short sequences (E value: 7e-70 for KX905134 vs. 3e-68 for LC193725).

Furthermore, the common detection of herpesvirus in equine populations using molecular approaches reinforces the complex nature of EHV infections and their widespread prevalence within equine populations, making them ubiquitous in horses, and not obligatorily associated with respiratory disease. Finally, the lack of detection of a representative number of reads or long contigs may indicate that they were not implicated in the development of respiratory disease among the sampled horses. Otherwise, the timing of sample collection, relative to the clinical course, may not have been optimal for the detection of these viruses, as some viral infections may have a short excretion period and, therefore, may no longer be detectable by direct diagnostic methods. It is worth considering that many bacteria can cause both chronic and acute respiratory infections, some of which may contribute to the development of asthma; these infections are often underlying causes of respiratory illnesses in horses. Finally, in one horse belonging to group B, the presence of the 753 bp sequence referring to EHV-1 transactivation of immediate early genes could be associated with viral replication and could regulate gene expression of the replication cycle of herpesviruses. Herpesviruses, like other DNA viruses, have a highly regulated replication cycle divided into different stages: immediate early (IE), early (E), and late (L) genes. The transactivation of IE genes is the first step in this cycle. The transactivation of immediate early genes is a key event in orchestrating the sequential expression of viral genes and ensuring the efficient replication of herpesviruses within the host cell. Therefore, we can hypothesize that the detection of the viral sequence through NGS may be related to an early phase of viral reactivation, following a previous herpesvirus infection.

Since no other viral pathogens were found in any sample from either group, it is suggested that poor performance or exercise intolerance and the involvement of viral etiological agents seems not to be frequent in horses with respiratory issues associated with poor performance or exercise intolerance. However, the number of subjects included in the sample was small, and it would be necessary to expand the study population to evaluate the real correlation between this syndrome and viral infections of the lower respiratory tract.

## 5. Conclusions

The study confirmed that collateral examinations performed using bronchoalveolar lavage fluid recovery are, indeed, capable of providing indications for respiratory system issues, including for the diagnosis of respiratory disease related to poor performance or exercise intolerance, as evidenced by the cellular alterations present in the subjects included in group B. In the examined samples, using both molecular methods and state-of-the-art techniques, no presence of viral agents was detected. Although the number of subjects in the study is small, this suggests that the involvement of viral etiological agents is not frequently observed in subjects with respiratory issues associated with poor performance or exercise intolerance.

## Figures and Tables

**Table 1:** Viral target selected.

Virus	Primers	5′-3′ Sequence	Amplicon Length	Tm	References
Equine Rhinitis B	EQ_RINB_F1	TTTTGATGCTTCACATTCTCC	781	60	[16]
	EQ_RINB_R1	CGCTGTACCCTCGGTCCTACC			
	EQ_RINB_F2	CTTACTAYGAATGTGARGGGC	662		
	EQ_RINB_R2	GCCTCGGCGAGTGAAGAG			
EHV 5	EHV5_F	ATGAACCTGACAGATGTGCC	290	56	[18]
	EHV5_R	CACGTTCACTATCACGTCGC			
Bosavirus	Bosavirus_qF	ACAGTCAAGGCAGGAGAAGG	137	60	[10]
	Bosavirus_qR	GGCTCCGTCTGGTTTTCTCA			
Horse parvovirus CSF	Horse_CSF_qF	CAAGGCTTTGGACAAACGGG	108	60	
	Horse_CSF_qR	TTGTTAGCACATGCGTTCCC			
Equicopivirus	EQCopivirus_qF	TCGCCCAGATCGTTGAGAAC	138	60	
	EQCopivirus_qR	AGCTGCTGTCTCCTGTTGTC			
EHV 1	EHV1_F1	TCTACCCCTACGACTCCTTC	1880	56	[15]
	EHV1_R1	GCTTTCTTTTCCTGCTTTT			
	EHV1_F2	CTTTAGCGGGTGATGTGGAAT	1275		
	EHV1_R2	TCTATTGCGTTTGCTATGCT			
EHV 4	EHV4_F1	TCTACCCCTACGACTCCTTC	932	56	
	EHV4_R1	TCCTGGTTGTTATTGGGTAT			
	EHV4_F2	TGTTTCCGCCACTCTTGACG	580	67	
	EHV4_R2	ACTGCCTCTCCCACCTTACC			
Equine GAPDH	AF083897_F	GGTGAAGGTCGGAGTAAACG	106	60	[17]
	AF083897_R	AATGAAGGGGTCATTGATGG			

**Table 2:** Age, breed, clinical signs, mucus score, respiratory endoscopy, BALF cytology alterations, and diagnoses reported for the 9 horses included in group B.

	Age	Breed	Clinical Signs	Mucus Score	Respiratory Endoscopy	BALF Cytology	Diagnosis
1	2y	TB	Sporadic cough at the beginning of the training session, poor performance.	3/5	Laryngeal lymphoid hyperplasia grade 3/4	Lymphocytes < 40%, neutrophils > 5%.	MEA
2	4y	TB	Sporadic cough at the beginning of the training session, poor performance.	3/5		Macrophages > 70%, lymphocytes < 40%, neutrophils > 5%, emosiderophages > 20%.	MEA and EIPH
3	3y	TB	Sporadic cough with cold temperature, poor performance.	2/5	Normal	Macrophages < 60%, eosinophils > 1%.	MEA
4	18y	Saddlehorse	Exercise intolerance, increased respiratory rate and respiratory effort.	3/5	Normal	Neutrophils > 20%.	SEA
5	9y	Trotter	Cough at rest, nasal discharge.	2/5	Normal	Neutrophils > 5%, macrophages < 60%.	MEA
6	9y	Trotter	Cough at rest.	3/5	Normal	Neutrophils > 5%, macrophages < 60%.	MEA
7	6y	Trotter	History of clinical signs in the last month.	2/5	Normal	Neutrophils > 5%, eosinophils > 1%.	MEA
8	10y	Trotter	History of clinical signs in the last month.	3/5	Normal	Neutrophils > 5%, macrophages < 60%, eosinophils > 1%.	MEA
9	12y	Trotter	History of clinical signs in the last month.	2/5	Normal	Macrophages < 60%, eosinophils > 1%.	MEA

## Data Availability

The data are available on request to the corresponding author.
